# Editorial: Dynamic vision test application and mechanism

**DOI:** 10.3389/fnins.2024.1456810

**Published:** 2024-07-22

**Authors:** Andrea Pavan, Yuexin Wang, Xuemin Li, Xiaoyu Liu

**Affiliations:** ^1^Department of Psychology, University of Bologna, Bologna, Italy; ^2^Department of Ophthalmology, Peking University Third Hospital, Beijing, China; ^3^Key Laboratory of Biomechanics and Mechanobiology, Ministry of Education, Beijing Advanced Innovation Center for Biomedical Engineering, School of Biological Science and Medical Engineering, Beihang University, Beijing, China; ^4^State Key Laboratory of Virtual Reality Technology and Systems, Beihang University, Beijing, China

**Keywords:** dynamic vision, dynamic vision test, motion vision, eye movement, sports vision

Dynamic visual acuity (DVA) is crucial for identifying visual targets amidst relative movements between subjects and objects, playing a vital role in everyday activities such as sports, and driving. Unlike static visual acuity, DVA is influenced by movement speed, patterns, and eye movements (Palidis et al., 2017). Evaluating DVA is very important in clinical settings as it reflects visual quality in real-life scenario. Research has demonstrated that DVA is essential for various reasons, including its significance in understanding vestibular impairments, enhancing performance in sports and traffic scenarios, and serving as a biometric indicator for astronaut performance and safety (Quevedo et al., 2018; Wu et al., 2021; Chen et al., 2023; Waisberg et al., 2023). The objective of this Research Topic was to gather original research and review existing literature on DVT across various clinical and occupational contexts. The aim was to encourage the use of DVA in diagnosing a wider range of diseases, evaluating conditions, and conducting occupational screenings.

## Research 1

Wang et al. (see also Wang et al., 2024 for corrigendum) investigated the relationship between patient-reported visual disturbances (e.g., glare, halos, starbursts, double vision, ghosting) and dynamic visual acuity in myopic patients following corneal refractive surgery. Their study included 95 myopic patients who underwent various types of corneal refractive surgeries and examined how these post-surgery disturbances impacted the ability to identify moving visual targets. These patients' DVA was measured using moving optotypes at different speeds (40 and 80 dps) 3 months post-surgery. The most frequently reported visual disturbance was the fluctuation in vision, occurring in 70.5% of the patients. The authors found significant correlations between worse DVA at 80 dps and the presence of haloes and difficulty in judging distance. The study revealed that visual disturbances, especially halos and difficulty in judging distance, significantly affected DVA, particularly at high speed. This study highlighted the importance of DVA tests and patient-reported quality of vision assessments in myopic patients following corneal refractive surgery.

## Research 2

Li et al. aimed to compare the DVA following implantation of toric trifocal or bifocal intraocular lenes (IOLs) in age-related cataract patients and to investigate potential factors associated with post-operative DVA. Advancements in surgical techniques and IOL design have transformed cataract surgery into a procedure that enhances quality of life by providing a full range of vision and reducing dependence on spectacles. Patients with age-related cataract and corneal astigmatism of at least 0.75 D were included. Following cataract surgery, patients were assessed for uncorrected and corrected distance, intermediate, and near static visual acuity, as well as uncorrected and correct distance DVA at 20, 40, and 80 dps. The study found that patients with toric bifocal IOL implantation had better corrected distance DVA at high speed than those with toric trifocal IOL. There were no significant differences in uncorrected and corrected distance DVA at low speeds between the two toric IOL groups. The study also found that age and postoperative static vision were crucial factors influencing DVA outcomes. In conclusion, while toric trifocal IOLs are better for intermediate static visual acuity, toric bifocal IOLs excel in high-speed distance DVA. The results underline the importance of considering age and static vision when selecting IOLs for cataract patients and suggest that DVA could be a valuable indicator for evaluating functional vision post-implantation.

## Research 3

Cataract surgery improves both static and dynamic vision. Wu et al. aimed to investigate the influence of corneal higher-order aberrations (HOAs) on DVA following cataract surgery. The study included 27 patients with 45 eyes who had undergone cataract surgery. Postoperative monocular DVA was assessed at different velocities (20, 40, and 80 dps) 1 month after surgery. Corneal HOAs were measured using Scheimpflug-based corneal topography. Significant differences in DVA were observed at different velocities, with 20 dps DVA significantly better than 40 and 80 dps DVA, and no significant difference between 40 and 80 dps DVA. Larger corneal HOAs, particularly coma and trefoil aberrations, negatively impacted high-speed DVA after cataract surgery, while spherical aberrations did not. This study provided insights into the relationship between DVA and high-order aberration and suggested considerations for surgical planning and patient counseling in cataract patients with dynamic vision demands.

## Research 4

The study of Liu et al. investigated the impact of different types of intraocular lenses (IOLs) on visual performance and eye movements in post-cataract surgery patients. The study compares blue light-filtering IOLs (yellow-tinted) with ultraviolet (UV) light-filtering IOLs (clear) by assessing visual performance and eye movements during everyday tasks such as non-social object search, face recognition, and reading. The findings indicate that while both IOLs effectively restore static visual function, subtle differences exist in eye movement patterns. Patients with blue light-filtering IOLs exhibited slightly increased fixation counts and longer search times in non-social object tasks, but no significant differences in face recognition and reading tasks were observed between two IOL types. These findings could help inform personalized IOL choices for patients, considering the slight variations in social visual performance and eye movement patterns associated with each IOL type.

## Conclusions

These studies collectively emphasize the significance of DVA testing in evaluating postoperative visual performance. They highlight the importance of considering factors such as age and static vision in assessing DVA outcomes. The research proposes that DVA is a valuable indicator for assessing functional vision after refractive surgery. These insights are critical for surgical planning and patient counseling, facilitating more personalized IOL choices to meet the patients' needs.

## Author contributions

AP: Validation, Conceptualization, Writing – review & editing, Writing – original draft. YW: Writing – review & editing, Writing – original draft, Software, Investigation, Funding acquisition, Conceptualization. XLi: Writing – review & editing, Writing – original draft, Software, Investigation, Conceptualization. XLiu: Writing – review & editing, Writing – original draft, Software, Investigation, Conceptualization.
